# Adaptive Baseline Enhances EM-Based Policy Search: Validation in a View-Based Positioning Task of a Smartphone Balancer

**DOI:** 10.3389/fnbot.2017.00001

**Published:** 2017-01-23

**Authors:** Jiexin Wang, Eiji Uchibe, Kenji Doya

**Affiliations:** ^1^Integrated System Biology Laboratory, Department of System Science, Graduate School of Informatics, Kyoto University, Kyoto, Japan; ^2^Neural Computation Unit, Okinawa Institute of Science and Technology Graduate University, Onna-son, Okinawa, Japan; ^3^Department of Brain Robot Interface, ATR Computational Neuroscience Laboratories, Soraku-gun, Kyoto, Japan

**Keywords:** smartphone robot, reinforcement learning, EM-based policy search, non-linear motor control, vision-based control

## Abstract

EM-based policy search methods estimate a lower bound of the expected return from the histories of episodes and iteratively update the policy parameters using the maximum of a lower bound of expected return, which makes gradient calculation and learning rate tuning unnecessary. Previous algorithms like Policy learning by Weighting Exploration with the Returns, Fitness Expectation Maximization, and EM-based Policy Hyperparameter Exploration implemented the mechanisms to discard useless low-return episodes either implicitly or using a fixed baseline determined by the experimenter. In this paper, we propose an adaptive baseline method to discard worse samples from the reward history and examine different baselines, including the mean, and multiples of SDs from the mean. The simulation results of benchmark tasks of pendulum swing up and cart-pole balancing, and standing up and balancing of a two-wheeled smartphone robot showed improved performances. We further implemented the adaptive baseline with mean in our two-wheeled smartphone robot hardware to test its performance in the standing up and balancing task, and a view-based approaching task. Our results showed that with adaptive baseline, the method outperformed the previous algorithms and achieved faster, and more precise behaviors at a higher successful rate.

## Introduction

Policy search methods (Deisenroth et al., 2013) are often favored over value function-based reinforcement learning for complex robotic problems because of their amenability with high-dimensional continuous states and actions. Classical policy search methods are called policy gradient methods such as REINFORCE (Williams, 1992), which compute the gradient of the objective function with respect to policy parameters and update the parameters by stochastic gradient ascent. However, REINFORCE samples a random action from the stochastic policy at each time step. As a result, the gradient estimate has large variance even if the optimal baseline is subtracted (Zhao et al., 2012). To reduce the gradient’s variance, the Policy Gradients with Parameter-based Exploration (PGPE) (Sehnke et al., 2010) uses a deterministic policy and optimizes the parameters of a prior distribution of the deterministic policy parameters. Natural Evolution Strategy (NES) (Wierstra et al., 2014) uses the natural gradient to update a parameterized search distribution in the direction of higher expected fitness, which can be applied under the PGPE framework. Note that all of these methods need to tune the learning rate.

Another trend is EM-based policy search methods based on closed-form maximization of the lower bound of the objective function with respect to the parameters of exponential family distributions. Those methods are realized as simple reward-weighted updating rule and do not require gradient computation and learning rate parameter tuning. Such methods include EM-inspired reward-weighted regression (RWR) (Peters and Schaal, 2007), EM Policy learning by Weighting Exploration with the Returns (PoWER) (Kober and Peters, 2011), Fitness Expectation Maximization (FEM) (Wierstra et al., 2008), and EM-based Policy Hyperparameter Exploration (EPHE) (Wang et al., 2016). RWR first introduced the framework of EM-based reinforcement learning and reduced the problem of learning with immediate rewards to a RWR problem with an adaptive reward transformation for faster convergence (Peters and Schaal, 2007). However, RWR considered a stochastic policy using additive Gaussian noise to the action, which could have problems of the perturbations being averaged out and the risk of damage to a hardware system with high frequency noise. PoWER considered state-dependent exploration to address these problems and adjusted the exploration to stepwise, episode-wise, and even through a slowly varying form (Kober and Peters, 2011). FEM inherited the same EM-based policy search framework and considered a rank-based return transformation function. It also considered an online mechanism to update policy sample by sample than batch by batch. A forgetting factor was introduced to modulate the speed at which the search policy adapts to the current sample (Wierstra et al., 2008). EPHE assumed a prior distribution over the policy parameters inspired by PGPE and updated the hyperparameters by return weighted averaging. REPS (Peters et al., 2010) has the similar idea of EM-based methods for bounding two distributions. The difference is that it bounds the information loss measured by relative entropy between the observed data distribution and the data distribution generated by the new policy to update the policy parameters.

Other state-of-the-art policy search methods include Cross Entropy Methods (CEM) (Mannor et al., 2003), Covariance Matrix Adaptation-Evolutionary Strategy (CMAES) (Hansen and Ostermeier, 2001), Policy Improvement with Path Integral (PI^2^) (Theodorou et al., 2010), and PI^2^-CMAES (Stulp and Sigaud, 2012). CEM and CMAES explore in the policy parameter space directly, and they both update mean and covariance matrix of a multivariate Gaussian search distribution in a weighting scheme. CEM updates the diagonal covariance, while CMAES updates the full covariance matrix through incremental adaption along evolutionary paths. PI^2^ is a probability weighting method derived from first principles of optimal control, perturbs the parameter and collects rewards at every time step during exploratory policy execution, and updates the policy parameter weighted by the probability of the rewards. PI^2^-CMAES improved PI^2^ by updating the full covariance matrix.

Note that EPHE is equivalent to episodic PoWER and PI^2^ (Stulp and Sigaud, 2012; Abdolmaleki et al., 2015).

Concerning the weighting scheme for each rollout, RWR, PoWER, FEM, and EPHE required the returns to be non-negative and summed up to constants to implicitly resemble a proper distribution. PoWER implicitly realized the discarding rule, and FEM and EPHE discarded samples below a fixed baseline determined by users, which is similar to CEM. CMAES reweighted the samples similarly by truncating the sample size and a transformation to a convex shape. REPS reweighted the samples in an exponential transformation of the corresponding return.

In this work, we propose an adaptive baseline to discard worse samples below the average of the reward history and further examine the learning performances of different baselines including the mean and 1 and 2 SDs from the mean. We implemented this adaptive baseline with EPHE. We evaluated several baseline functions in three simulation benchmarks. The result showed that the adaptive baseline methods improved the performance over previous policy search methods, including PGPE, NES, and FEM, and outperformed the CMAES weighting scheme and REPS weighting scheme.

We further tested the adaptive baseline with the mean in two hardware experiments using our smartphone robot platform (Yoshida et al., 2012; Wang et al., 2013, 2014), an affordable high-performance desktop robotic platform for multi-agent research, educational, and hobby use. The actual hardware experiments required a smartphone balancer learning to stand up and balance and learning to approach a visual target while balancing. Previous PoWER was implemented in a real robot arm for playing a ball-in-a-cup task using imitation learning for parameter initialization (Kober and Peters, 2011). In our smartphone robot experiment of learning to stand up and balance and learning to approach a visual target, the hyperparameters were learned from scratch, and the robustness against the variability of initial states was required. Our results showed that the adaptive baseline method with the mean successfully achieved the two behaviors in hardware experiments. The analyses of the number of discarded samples and the distribution of returns showed that the choice of the baseline by the mean caused a steady decrease of the number of discarded samples with learning and a capability to select positive outliers in the early learning stage.

The details of EPHE and its improvement with adaptive baselines are described in Section “Method.” Simulation experiments and results are shown in Section “Simulation Experiments.” Hardware experiments and results are reported in Section “Hardware Experiments.” Conclusion and future works are in Section “Discussion.”

## Method

Here, we consider a standard discrete-time Markov Decision Process setup. At each time step *t*, the agent takes action *u_t_* based on current state *x_t_* according to policy π (*u_t_*|*x_t_*,θ) parameterized by vector θ. The dynamic environment makes a transition to next state *x_t_*_+ 1_ according to *p*(*x_t_*_+ 1_ |*x_t_, u_t_*) and gives scalar reward *r_t_*. The history trajectory is denoted by a state-action-reward sequence as *h* = [*x*_1_, *u*_1_, *r*_1_, …, *x_T_*, *u_T_*, *r_T_*, *x_T_*_+1_]. The goal is to find parameter θ that maximizes an objective function defined as the agent’s expected sum of reward:

$$J\left(\theta \right)=\underset{H}{\int }\;p\left(h|\theta \right)R\left(h\right)\mathrm{dh},$$

where *R*(h) is the return defined as the cumulative reward of sequence *h* and *p*(*h*/θ) is the probability of observing *h*. Under the Markovian environmental assumption, the probability of sequence *p*(*h*/θ) for the stochastic policy is given by:

(1) $$p\left(h|\theta \right)=p\left(x_{1}\right)\prod_{t=1}^{T}\;p\left(x_{t+1}|x_{t},u_{t}\right)\pi \left(u_{t}|x_{t},\theta \right)$$

where *p*(*x*_1_) is the initial state distribution. A standard stochastic policy search method like REINFORCE maximizes *J*(θ) by updating parameter θ based on the gradient of *J*(θ) with respect to θ. However, it is usually problematic to select an action from the stochastic policy at each time step in the robot control from the viewpoint of stability.

If the policy is deterministic, denoted by *u_t_* = π(*x_t_*, θ), Eq. 1 is modified as

$$p\left(h|\theta \right)=p\left(x_{1}\right)\prod_{t=1}^{T}\;p\left(x_{t+1}|x_{t},\pi \left(x_{t},\theta \right)\right),$$

and derivative ▽_θ_*p*(*h*|θ) for deterministic policies is given by Deisenroth et al. (2013),

$$\nabla_{\theta }p\left(x_{t+1}|x_{t},\pi \left(x_{t},\theta \right)\right)=\frac{\partial p\left(x_{t+1}|x_{t},u_{t}\right)}{\partial u_{t}}{\left.\frac{\partial u_{t}}{\partial \theta }\right|}_{u_{t}=\pi \left(x_{t},\theta \right)},$$

which requires the state transition probability to compute the derivative with respect to *u*. This suggests that the state transition probability should be given or estimated to compute the policy gradient, although a recent study (Silver et al., 2014) showed that the gradient for deterministic policies can be estimated by the help of the state-action value function.

### Policy Gradients with Parameter-Based Exploration (PGPE)

To optimize the deterministic policies, PGPE (Sehnke et al., 2010) considers their distribution with policy parameters θ sampled from a prior distribution defined by hyperparameter vector ρ. The PGPE’s goal is to maximize the objective function given by

$$J\left(\rho \right)=\underset{\Theta }{\int }\;\underset{\text{H}}{\int }\;p\left(h|\theta \right)p\left(\theta |\rho \right)R\left(h\right)\mathrm{dhd}\theta .$$

PGPE optimizes hyperparameter vector ρ by the stochastic gradient ascent, ρ ← ρ + α▽_ρ_*J*(ρ), where α is a learning rate and the gradient is given by

$$\nabla_{\rho }J\left(\rho \right)=\underset{\Theta }{\int }\;\underset{\text{H}}{\int }\;p\left(h|\theta \right)\left(R\left(h\right)-b\right)\nabla_{\rho }p\left(\theta |\rho \right)\mathrm{dhd}\theta ,$$

where *b* is a reward baseline to decrease the variance of the gradient estimator. The baseline is set as the average reward of the current rollout for simplicity, although it is not optimal (Hachiya et al., 2011). After the optimization of hyperparameter ρ, we can obtain the deterministic policy *u_t_* = π(*x_t_*, θ) with θ computed by the expectation of the prior distribution. Note that PGPE does not need to know the state transition probability to compute the gradient.

### EM-Based Policy Hyperparameter Exploration

Based on the PGPE setting, EPHE (Wang et al., 2016) adopts the idea from EM-based Policy Search (Peters and Schaal, 2007) of maximizing a lower bound of the objective function by a new parameter distribution given by hyperparameter vector ρ′. Using Jensen’s inequality under the assumption that *R*(*h*) is strictly positive, the log ratio of the two objective functions is lower bounded by

$$\begin{array}{llll} \text{log}\frac{J\left(\rho \prime \right)}{J\left(\rho \right)} & =\text{log}\underset{\Theta }{\int }\;\underset{\text{H}}{\int }\;\frac{R\left(h\right)p\left(h|\theta \right)p\left(\theta |\rho \right)}{J\left(\rho \right)}\frac{p\left(\theta |\rho \prime \right)}{p\left(\theta |\rho \right)}\mathrm{dhd}\theta \; & & \;\\ & \;\geq \underset{\Theta }{\int }\;\underset{\text{H}}{\int }\;\frac{R\left(h\right)p\left(h|\theta \right)p\left(\theta |\rho \right)}{J\left(\rho \right)}\text{log}\frac{p\left(\theta |\rho \prime \right)}{p\left(\theta |\rho \right)}\mathrm{dhd}\theta .\; & & \; \end{array}$$

Note that *R*(*h*)*p*(*h*|θ)*p*(θ|ρ)/*J*(ρ) can be interpreted as a probability density function *p*(*z*|ρ) where *z* = (*h*, θ), and we denote *f* (*z*,ρ) ≜ *p*(θ|ρ′)/*p*(θ|ρ) where the Jensen’s inequality follows

log∫f(z,ρ)p(z|ρ)dz≥∫p(z|ρ)logf(z,ρ)dz.

Since

$$\begin{array}{llll} \text{log}\frac{J\left(\rho \prime \right)}{J\left(\rho \right)} & =\text{log}\;J\left(\rho \prime \right)-\;\text{log}\;J\left(\rho \right)\; & & \;\\ & \;\geq \underset{\Theta }{\int }\;\underset{\text{H}}{\int }\;\frac{R\left(h\right)p\left(h|\theta \right)p\left(\theta |\rho \right)}{J\left(\rho \right)}\text{log}\frac{p\left(\theta |\rho \prime \right)}{p\left(\theta |\rho \right)}\mathrm{dhd}\theta ,\; & & \; \end{array}$$

the lower bound is defined by

(2) $$\text{log}\;J_{L}\left(\rho \prime \right)=\;\text{log}\;J\left(\rho \right)+\underset{\Theta }{\int }\;\underset{\text{H}}{\int }\;\frac{R\left(h\right)p\left(h|\theta \right)p\left(\theta |\rho \right)}{J\left(\rho \right)}\text{log}\frac{p\left(\theta |\rho \prime \right)}{p\left(\theta |\rho \right)}\mathrm{dhd}\theta .$$

To maximize the lower bound, the derivative of Eq. 2 with respect to ρ′ should equal 0

$$\nabla_{\rho \prime }\text{log}\;J_{L}\left(\rho \prime \right)=\underset{\Theta }{\int }\;\underset{\text{H}}{\int }\;\frac{R\left(h\right)p\left(h|\theta \right)p\left(\theta |\rho \right)}{J\left(\rho \right)}\nabla_{\rho \prime }\text{log}\frac{p\left(\theta |\rho \prime \right)}{p\left(\theta |\rho \right)}\mathrm{dhd}\theta =0.$$

Since *J*(ρ) is constant, this equation can be simplified as:

$$\underset{\Theta }{\int }\;\underset{\text{H}}{\int }\;R\left(h\right)p\left(h|\theta \right)p\left(\theta |\rho \right)\nabla_{\rho \prime }\text{log}\;p\left(\theta |\rho \prime \right)\mathrm{dhd}\theta =0.$$

When applying the sampling trick, suppose we sample *N* set of θ from *p*(θ|ρ) and generate *M* set of *h* from *p*(h|θ*^n^*), we have

$$\frac{1}{N}\sum_{n=1}^{N}\underset{H}{\int }\;R\left(h\right)p\left(h|\theta^{n}\right)\nabla_{\rho \prime }\text{log}\;p\left(\theta^{n}|\rho \prime \right)\mathrm{dh}=0,$$

and

$$\frac{1}{M}\sum_{m=1}^{M}\;\frac{1}{N}\sum_{n=1}^{N}\;R\left(h_{m}^{n}\right)\nabla_{\rho \prime }\text{log}\;p\left(\theta^{n}|\rho \prime \right)=0.$$

For simplicity, if we generate *N* set of θ, and for each set of θ, we sample only one history trajectory, we have

(3) $$\frac{1}{N}\sum_{n=1}^{N}\;R\left(h^{n}\right)\nabla_{\rho \prime }\text{log}\;p\left(\theta^{n}|\rho \prime \right)=0.$$

If *p*(θ|ρ′) is represented by an exponential family distribution, the update rule is given by a closed form. In particular, *p*(θ|ρ′) is given by a product of independent Gaussian distributions $N\left(\theta_{i}|\eta_{i}^{\prime },{\sigma_{i}^{\prime }}^{2}\right)$ for each parameter θ*_i_* in θ, where the hyperparameters are given by $\rho \prime ={\left[\eta_{1}^{\prime },\ldots ,\eta_{L}^{\prime },\sigma_{1}^{\prime },\ldots ,\sigma_{L}^{\prime }\right]}^{T}$ and *L* is the dimensionality of policy parameter θ. The log derivatives of *p*(θ|ρ′) with respect to $\eta_{i}^{\prime }$ and $\sigma_{i}^{\prime }$ are, respectively, computed as

(4) $$\nabla_{\eta_{i}^{\prime }}\text{log}\;p\left(\theta |\rho^{\prime }\right)=\frac{\theta_{i}-\eta_{i}^{\prime }}{{\sigma_{i}^{\prime }}^{2}},$$

(5) $$\nabla_{\sigma_{i}^{\prime }}\text{log}\;p\left(\theta |\rho^{\prime }\right)=\frac{{\left(\theta_{i}-\eta_{i}^{\prime }\right)}^{2}-{\sigma_{i}^{\prime }}^{2}}{{\sigma_{i}^{\prime }}^{3}}.$$

Substituting Eqs 4 and 5 into Eq. 3 yields

$$\begin{array}{llll} \eta_{i}^{\prime } & =\frac{\sum_{n=1}^{N}\left[R\left(h^{n}\right)\theta_{i}^{n}\right]}{\sum_{n=1}^{N}R\left(h^{n}\right)},\; & & \;\\ \sigma_{i}^{\prime } & =\sqrt{\frac{\sum_{n=1}^{N}\left[R\left(h^{n}\right){\left(\theta_{i}^{n}-\eta_{i}^{\prime }\right)}^{2}\right]}{\sum_{n=1}^{N}R\left(h^{n}\right)}}.\; & & \; \end{array}$$

**Figure 1:**
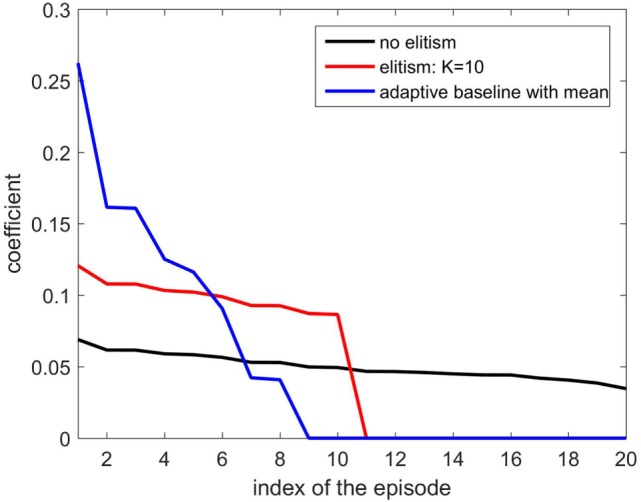
**Weight comparison of EM-based Policy Hyperparameter Exploration (EPHE)-AB-mean, EPHE-K, and no elitism methods**.

In our previous EPHE implementation (Wang et al., 2016), we showed a *K*-elite mechanism (hereafter EPHE-K), in which we selected the *K*-best parameters to discard bad samples for updating the hyperparameters and improved the learning process. However, this requires a new parameter tuning. We denote the returns collected at the current iteration step as ${\left\{R\left(h^{n}\right)\right\}}_{n=1}^{N}$ and propose an adaptive baseline using the mean value of the returns:

(6) $$R_{b}\left(h^{n}\right)=\text{max}\;\left(0,R\left(h^{n}\right)-\text{mean}\;\left({\left\{R\left(h^{n}\right)\right\}}_{n=1}^{N}\right)\right)\;,$$

where $\text{mean}\left({\left\{R\left(h^{n}\right)\right\}}_{n=1}^{N}\right)=\sum_{n=1}^{N}\;R\left(h^{n}\right)∕N.$ Note that *R_b_*(h) should be kept non-negative to resemble an (improper) probability distribution to weight the parameters. Hereafter, we call the adaptive baseline by Eq. 6 the EPHE-AB-mean. Figure 1 illustrates an example of weighting by the EPHE-AB-mean, EPHE-K, and EPHE with no elitism. Suppose that we have 20 returns following a Gaussian distribution and sort them in descending order as ${\left\{R^{i}\right\}}_{i=1}^{20}.$ Then, weighting coefficients $R^{i}∕\sum_{i=1}^{20}\;R^{i}$ are computed for the EPHE-AB-mean, EPHE-K(=10), and the no-elite methods. Unlike EPHE-K (=10), the EPHE-AB-mean enhances the differences of the returns.

In the same way, we consider the adaptive baseline by replacing the mean operator with the m-SD from the mean operator defined by

$$\text{mean}-\text{mstd}\;\left({\left\{R\left(h^{n}\right)\right\}}_{n=1}^{N}\right)=\text{mean}\;\left({\left\{R\left(h^{n}\right)\right\}}_{n=1}^{N}\right)+\text{m}\times \text{std}\;\left({\left\{R\left(h^{n}\right)\right\}}_{n=1}^{N}\right).$$

The EPHE-AB algorithm is summarized as follows:

**Algorithm:** EPHE-AB: EM-based Policy Hyperparameter Exploration with Adjusted Baseline.

**Input:**
Initialize policy hyper parameters η and σ
**Repeat:**
Perform *N* episodes and sample trajectories:
for each episode *n*
draw $\theta_{i}^{n}∼\;N\left(\eta_{i},\sigma_{i}^{2}\right)$ for all *i*
evaluate *R*(h*^n^*)
calculate
$R_{b}\left(h^{n}\right)=\text{max}\;\left(0,R\left(h^{n}\right)-\;\text{mean}\;\left({\left\{R\left(h^{n}\right)\right\}}_{n=1}^{N}\right)\right)$
Update
$\eta_{i}=\frac{\sum_{n=1}^{N}\left[R_{b}\left(h^{n}\right)\theta_{i}^{n}\right]}{\sum_{n=1}^{N}R_{b}\left(h^{n}\right)}$
$\sigma_{i}=\sqrt{\frac{\sum_{n=1}^{N}\left[R_{b}\left(h^{n}\right){\left(\theta_{i}^{n}-\eta_{i}\right)}^{2}\right]}{\sum_{n=1}^{N}R_{b}\left(h^{n}\right)}}$
**Until** Convergence

### Policy Learning by Weighting Exploration with the Returns

EM-based Policy Search (Peters and Schaal, 2007) has the lower bound of the log-expected return defined from Jensen’s inequality as

$$\text{log}\;J\left(\theta \prime \right)\geq \underset{H}{\int }\;\frac{R\left(h\right)p\left(h|\theta \right)}{J\left(\theta \right)}\text{log}\frac{p\left(h|\theta \prime \right)}{p\left(h|\theta \right)}\mathrm{dh}+\text{log}\;J\left(\theta \right)\equiv \text{log}\;J_{L}\left(\theta \prime \right)$$

where the current policy parameter θ is matched with a new policy parameter θ′. Furthermore, the log-derivative of the lower bound with respect to the new policy parameter is

$$\nabla_{\theta \prime }\text{log}\;J_{L}\left(\theta \prime \right)=\underset{H}{\int }\;p\left(h|\theta \right)R\left(h\right)\nabla_{\theta \prime }\text{log}\;p\left(h|\theta \prime \right)\mathrm{dh}.$$

With the sampling trick, it can be approximated as

(7) $$\nabla_{\theta \prime }\text{log}\;J_{L}\left(\theta \prime \right)\approx \frac{1}{N}\sum_{n=1}^{N}\sum_{t=1}^{T}\nabla_{\theta \prime }\text{log}\;\pi \;\left(u_{t}^{n}|x_{t}^{n},\theta \prime \right)R\left(h_{t}^{n}\right)$$

where *T* is the number of time steps, and *N* is the number of episodes.

In particular, PoWER (Kober and Peters, 2011) considered a structured state-dependent exploration, where a stochastic policy π(*u_t_*|*x_t_*, θ) is defined as

$$\begin{array}{llll} & u_{t}∼\pi \left(u_{t}|x_{t},\theta \right)={\left(\theta +\varepsilon \right)}^{T}\Phi \left(x_{t}\right),\; & & \;\\ & \varepsilon^{T}\Phi \left(x_{t}\right)∼\text{N}\left(0,\Phi {\left(x_{t}\right)}^{T}\Sigma \Phi \left(x_{t}\right)\right).\; & & \; \end{array}$$

Note that Φ(*x_t_*) is the basis function vectors. Substituting it into Eq. 7 yields the following update rules:

$$\begin{array}{ll} \theta \prime & =\theta \prime +\left(\frac{1}{N}\sum_{n=1}^{N}\sum_{t=1}^{T}W\left(x_{t}\right)R\left(h_{t}^{n}\right)\right)-1\times \left(\frac{1}{N}\sum_{n=1}^{N}\sum_{t=1}^{T}W\left(x_{t}\right)\varepsilon_{t}R\left(h_{t}^{n}\right)\right)\\ \Sigma \prime & ={\left(\frac{1}{N}\sum_{n=1}^{N}\sum_{t=1}^{T}R\left(h_{t}^{n}\right)\right)}^{-1}\left(\frac{1}{N}\sum_{n=1}^{N}\sum_{t=1}^{T}\varepsilon_{t}\varepsilon_{t}^{T}R\left(h_{t}^{n}\right)\right) \end{array}$$

where *W* (*x_t_*) = Φ(*x_t_*)Φ(*x_t_*)*^T^*(Φ(*x_t_*)*^T^*ΣΦ(*x_t_*))^−1^.

Note that in episodic case, the update rules are simplified as follows:

$$\begin{array}{llll} \theta \prime & =\theta \prime +{\left(\sum_{n=1}^{N}\;R\left(h^{n}\right)\right)}^{-1}\left(\sum_{n=1}^{N}\;\varepsilon \;R\left(h^{n}\right)\right)\; & & \;\\ \Sigma \prime & ={\left(\sum_{n=1}^{N}\;R\left(h^{n}\right)\right)}^{-1}\left(\sum_{n=1}^{N}\;\varepsilon \varepsilon^{T}R\left(h^{n}\right)\right).\; & & \; \end{array}$$

Consequently, it is shown that the original EPHE is equivalent to the episodic PoWER although the hyperparameters are not introduced in the episodic PoWER. The adaptive baseline can also be equipped to the episodic PoWER.

## Simulation Experiments

In this section, we compare EPHE-AB-mean with EPHE with CMAES weighting scheme (EPHE-CW) (Hansen and Ostermeier, 2001), EPHE with REPS weighting scheme (EPHE-RW) (Peters et al., 2010; Abdolmaleki et al., 2015), EPHE-K, PGPE (Sehnke et al., 2010), NES (Wierstra et al., 2014), and FEM (Peters and Schaal, 2007) in three simulation experiments. We also tested EPHE-AB with the baselines of the mean, 1 and 2 SDs from the mean. For each method we used *N* = 20 trajectories to update one set of parameters. We selected *K* = 10 to obtain the elite parameters for updating in EPHE-CW, EPHE-RW, and EPHE-K. The results were taken by averaging 20 independent runs. We plotted the learning curves of the average and the SE of the cumulative returns and the number of discarded samples against the iterations of the parameter updating.

The weights of EPHE-CW are computed by ${\text{w}}_{k}=\text{ln}\frac{K+1}{2}-\ln\;k,\;\text{ for}\;k=1,\ldots ,\;K.$

The weights of EPHE-RW are computed by ${\text{w}}_{k}=\text{exp}\left(\frac{R\left(h^{k}\right)}{\eta }\right),$ where η is the temperature parameter obtained by optimizing the dual function *g*(η), such that η > 0 (Peters et al., 2010; Kupcsik et al., 2013; Abdolmaleki et al., 2015)

$$g\left(\eta \right)=\eta \varepsilon +\eta \text{log}\;\left(\sum_{k=1}^{N}\frac{1}{N}\text{exp}\frac{R\left(h^{k}\right)}{\eta }\right)$$

and ε is the upper bound of KL divergence set as 0.1. We use the function fminunc in MATLAB to obtain the optimal η. Note that EPHE with REPS weighting scheme is equivalent to episodic REPS (Kupcsik et al., 2013).

### Pendulum Swing Up with Limited Torque

The target of this non-linear control task is to swing up the pendulum to an upright position where it stays for as long as possible (Doya, 2000). See Section “Simulation Setup” in Appendix for the details of the simulation setup. We used 16 × 16 radial basis functions to represent the two-dimensional state variables, the angle, and the angular velocity of the pendulum: $x={\left[\varphi ,\dot{\varphi }\right]}^{T}.$ We used the normalized radial basis function defined by

$$\Phi_{k}\left(\text{x}\right)=\frac{{e^{-∥s_{k}^{T}\left(x-c_{k}\right)∥}}^{2}}{{\sum_{k=1}^{K}e^{-∥s_{k}^{T}\left(x-c_{k}\right)∥}}^{2}}$$

where *k* is the index of the radial basis functions and *s_k_* and *c_k_* are the size and center of the *k*-th basis function. The action is the torque applied to the pendulum $u=5\;\tanh\;\left(\theta^{T}\Phi \left(\text{x}\right)\right)$ with maximum torque 5 [N⋅m], where **θ** is the policy parameter and Φ(***x***) is the basis function vector. A strictly positive return for one history is given by

(8) $$R\left(h\right)=\sum_{t=1}^{T}\text{exp}\;\left(-x_{t}^{T}Qx_{t}-u_{t}^{T}Ru_{t}\right).$$

where *Q* and *R* are the quadratic penalty matrices determined by the users. Here, we used *Q* = *I*_2_ and *R* = 1, where *I*_2_ is the 2-dimensional identity matrix. Note that a linear state feedback policy failed to achieve the task because the maximum torque was smaller than the maximal load torque (Wang et al., 2014). The system starts from initial state ${\text{x}}_{\text{0}}=\left[\varphi_{0},0\right],$ where φ_0_ was randomly selected from (−π, π] [rad], and terminated when $|\dot{\varphi }|\geq 4\pi$ [rad/s]. The sampling rate was 0.02 [s] for each time step and the maximum time steps were 1,000 (=20 [s]) for one episode. The initial hyper parameters were $\eta_{\text{0}}=\text{0},$ $\sigma_{\text{0}}=\text{1}$ for each algorithm. The best learning rates for PGPE and NES were α_η_ = 10^−3^ and α_σ_ = 10^−4^ which were selected, respectively, from sets {10^−2^, 10^−3^, 10^−4^, 10^−5^} and {10^−2^, 10^−3^, 10^−4^, 10^−5^}. The forgetting rate for FEM is 0.05.

**Figure 2:**
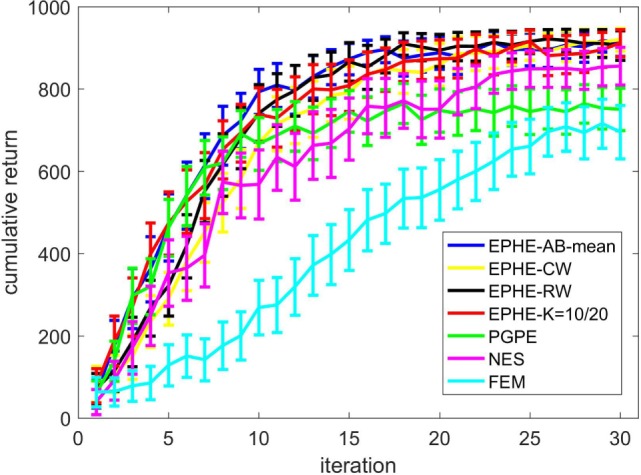
**Learning curves of EM-based Policy Hyperparameter Exploration (EPHE)-AB with baseline of mean, EPHE-CW, EPHE-RW, EPHE-K, PGPE, Natural Evolution Strategy, and Fitness Expectation Maximization in pendulum swing up task**.

Figure 2 shows the EPHE-AB performance with the baseline of mean, EPHE-CW, EPHE-RW, EPHE-K, PGPE, and NES with the best learning rate and FEM. The error bars show 1 SD over 20 independent runs. There was no significant difference between EPHE-AB and EPHE-K, but they learned faster and achieved better performance than FEM and gradient based PGPE and NES after 10 iterations.

**Figure 3:**
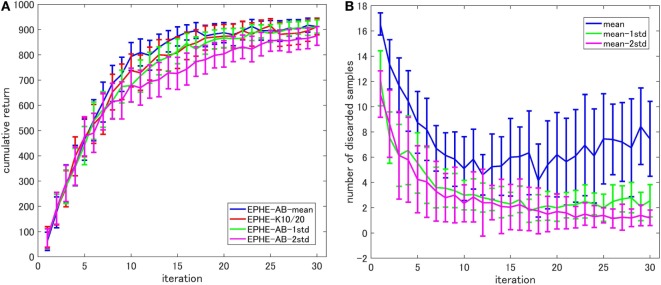
**Effects of baseline functions in pendulum swing up task: (A) learning curves and (B) number of discarded samples**.

Figure 3 shows the EPHE-AB performance with different baselines. There are no significant differences among the fixed baseline and the baselines of mean and the 1 SD from it. Figure 3B shows the number of discarded samples against the iterations, and we found that in the early stage of learning, the baseline with mean discarded most of the samples while the other three discarded about 60% of them.

### Cart-Pole Balancing

In this task, the agent aims to maximize the length of time that a movable cart is balancing a pole upright in the center of a track (Riedmiller et al., 2007). The state variables are the position and the velocity of the cart on the track and the angle and the angular velocity of the pole: $\text{x}={\left[x,ẋ,\;\varphi ,\;\dot{\varphi }\right]}^{T}.$ The action is the force applied to the cart given by a linear parameterized policy where $u=\theta^{T}\text{x}.$ See Section “Simulation Setup” in Appendix for the details. We added Gaussian white noise with SDs of 0.001 [rad/s] and 0.01 [m/s] to the dynamics. The system starts within a random position and a random angle inside [−0.2, +0.2] [rad], and [−0.5, +0.5] [m] until it reaches the target region of [−0.05, +0.05] [rad] and [−0.05, +0.05] [m], and terminates at |x|≥2.4 [m], and |θ|≥0.7 [rad]. The sampling rate was 0.02 [s] for each time step and the maximum time steps were 1,000 (=20 [s]) for one episode. The strictly positive return was the same as Eq. 8. Here, we used *Q* = *I*_4_ and *R* = 1. The initializations of the hyperparameters for PGPE and EPHE were determined by reasonable prior knowledge, which indicates a certain distance from the optimal parameters. The initial parameters were $\sigma_{\text{0}}=\text{35}$ for EPHEs, and FEM and $\sigma_{\text{0}}=\text{5}$ for PGPE and NES according to our previous study (Wang et al., 2016). The best learning rates of PGPE and NES were α_η_ = 10^−4^ and α_σ_ = 10^−5^ which were selected, respectively, from sets {10^−2^, 10^−3^, 10^−4^, 10^−5^} and {10^−2^, 10^−3^, 10^−4^, 10^−5^}. The forgetting rate for FEM is 0.1.

**Figure 4:**
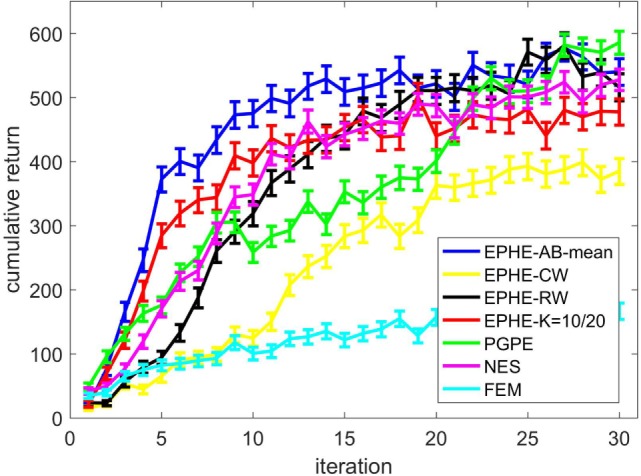
**Learning curves of EM-based Policy Hyperparameter Exploration (EPHE)-AB with baseline of mean, EPHE-CW, EPHE-RW, EPHE-K, PGPE, Natural Evolution Strategy, and Fitness Expectation Maximization in cart-pole balancing task**.

Figure 4 shows the EPHE-AB performance with a baseline of mean, EPHE-CW, EPHE-RW, EPHE-K, PGPE, and NES with the best learning rate and FEM. The error bars show 1 SD over 20 independent runs. The proposed method learned faster and achieved better performance after 10 iterations than the other algorithms. PGPE and NES with the best learning rate learned slowly, but they achieved almost the same performance as the best performance at the end of the iterations.

**Figure 5:**
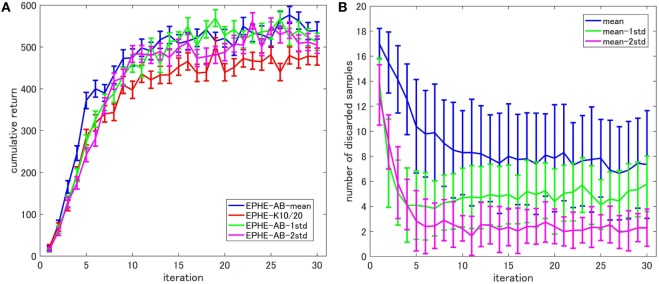
**Effects of baseline functions in cart-pole balancing task: (A) learning curves and (B) number of discarded samples**.

Figure 5 shows the EPHE-AB performance with different baselines (Figure 5A) and the number of discarded samples against the iterations (Figure 5B). There are no significant differences among the baselines of mean, 1 and 2 SDs from it. However, the adaptive baselines performed better than the fixed baseline. Figure 5B suggests that in the early stage of learning, the baseline with mean discarded most of the samples while the other three discarded about 70% of them.

### Standing Up and Balancing of Two-Wheeled Smartphone Robot

Next, we used a simulator of a two-wheeled smartphone robot (Wang et al., 2016). The agent is required to start moving from a resting angle of 60°, bounce with the bumper to stand up and finally balance itself. We considered only the sagittal plane behaviors for simplicity. The state variables are the body’s tilting angle and angular velocity, and the wheel’s rotating angle and angular velocity where $\text{x}={\left[\varphi ,\dot{\varphi },\;\vartheta ,\;\dot{\vartheta }\right]}^{T}.$ Control input *u* is the motor torque applied to the left and right wheels. See Section “Simulation Setup” in Appendix for details of the equation of motion. The cumulative reward is the same as in Eq. 8.

**Figure 6:**
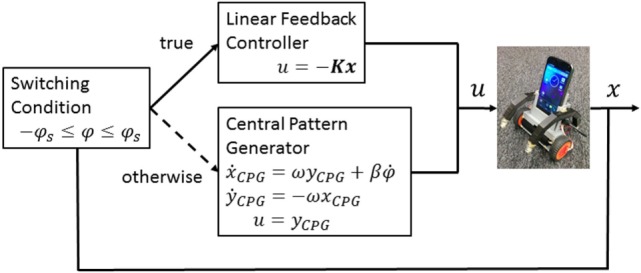
**Switching control architecture for standing up and balance of a smartphone robot**.

We adopted the switching framework shown in Figure 6. If the tilting angle of the robot body is within the range of $\left[-\varphi_{s},\varphi_{s}\right],$ we selected a linear feedback stabilizer *u* = −*Kx*, where $K=\left[k_{\varphi },k_{\dot{\varphi }},k_{\vartheta },k_{\dot{\vartheta }}\right]$ is a feedback gain vector, to achieve balancing. Otherwise, the central pattern generator (CPG)-based destabilizer is applied, defined by:

(9) $${ẋ}_{\mathrm{CPG}}=\omega y_{\mathrm{CPG}}+\beta \dot{\varphi },\;{ẏ}_{\mathrm{CPG}}=-\omega x_{\mathrm{CPG}},$$

where *x_CPG_* and *y_CPG_* are the CPG state and ω and β are its parameters. The control signal is given by *u* = *y_CPG_*. The policy parameters are the four-dimensional control gain vectors for the linear stabilizer, the switching threshold, and two CPG parameters: $\theta ={\left[k_{\varphi },k_{\dot{\varphi }},k_{\vartheta },k_{\dot{\vartheta }},\varphi_{s},\omega ,\beta \right]}^{T}.$ We also added Gaussian white noise with a SD of 0.01 to the system observation.

The simulation had a 0.02 [s] sampling rate for each step. The agent learned one episode within a maximum of 1,000 steps (=20 [s]). We initialized hyperparameters $\eta_{\text{0}}$ and $\sigma_{\text{0}}$ based on the prior knowledge we obtained (Wang et al., 2013). $\sigma_{\text{0}}$ for PGPE and NES was smaller than EPHE and FEM based on previous work (Wang et al., 2016), because PGPE uses small initial variance to approximate the gradient more precisely while EPHE uses large initial variance to explore the parameter space. The forgetting rate for FEM was 0.2.

**Figure 7:**
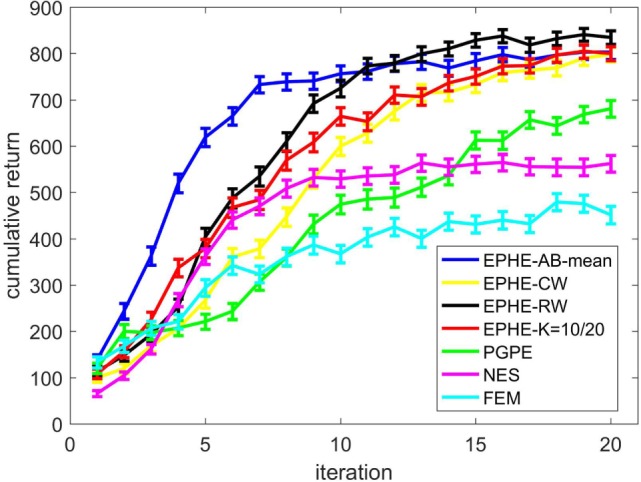
**Learning curve of EM-based Policy Hyperparameter Exploration (EPHE)-AB with mean, EPHE-CW, EPHE-RW, EPHE-K, PGPE, Natural Evolution Strategy, and Fitness Expectation Maximization in a standing up and balancing simulation of smartphone robot**.

Figure 7 shows the learning performance. The error bars show 1 SD over 20 independent runs. EPHE-AB with the mean learned faster than the other four algorithms and achieved a more reliable performance after seven iterations.

**Figure 8:**
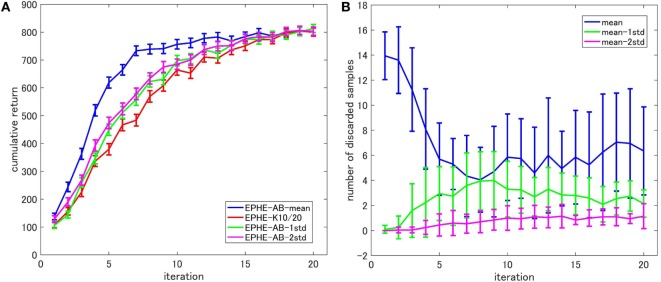
**Effects of baseline functions in a standing up and balancing simulation of smartphone robot task: (A) learning curves and (B) number of discarded samples**.

Figure 8 shows the EPHE-AB performance with different baselines (Figure 8A) and the number of discarded samples against the iterations (Figure 8B). The baseline of mean outperformed the others and the adaptive baselines performed better than the fixed baseline. Figure 8B suggests that in the early stage of learning, the baseline with mean discarded most of the samples and had a steady decrease during the learning. The baseline with 1 and 2 SDs from the mean preserved most of the samples due to a large SD of returns based on the reward function.

## Hardware Experiments

### Overview of Two-Wheeled Smartphone Robot

**Figure 9:**
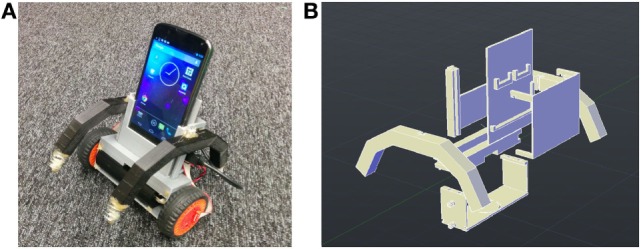
**Smartphone robot: current version (A) and 3D chassis (B)**.

Figure 9A shows the current version of our smartphone balancer. Its chassis is designed as an assembly kit for convenient composition and modification (Figure 9B). The smartphone rests in the middle slot, a battery is clipped to the bottom container, two wheels are inserted into Lego cross sticks extruding from the body side, and the IOIO board (the microcontroller chip) and break-out board slide into slots on the back. By sliding in two spring arms on both sides of the robot body, it becomes a spring-armed balancer that can achieve various standing up, balancing, and approaching behaviors. See more robot construction details in Section “Robot Construction” in Appendix.

### Standing Up and Balancing Task

#### Experimental Setting

This is a real hardware implementation of the simulation task in Section “Standing Up and Balancing of Two-Wheeled Smartphone Robot.” The balancer is required to learn to get off the floor and remain balancing in the upright region. It is equipped with an elastic bumper with a 0.8869 [N/mm] spring coefficient and an initial resting angle: φ_0_=25 [deg]. The state variables are the tilting angle and angular velocity of the body obtained from the sensor fusion of the gyroscope and accelerometer inside the smartphone, and the angular velocity of the left and right wheels from the rotary encoder inside the wheel: $\text{x}={\left[\varphi ,\dot{\varphi },\;{\dot{\vartheta }}_{L},{\dot{\vartheta }}_{R}\right]}^{T}.$ We eliminated the rotating angle of the wheels due to the accumulated error of the rotary encoder. Since we considered only the sagittal plane behaviors, the same control signal was sent to both wheels. The reward function was +1 when the tilting angle was inside [−5°, 5°] and the angular velocity of the robot body was inside [−173, 173] [deg/s], and otherwise it was 0.

We used the same control architecture in Figure 6. We fixed φ*_s_* = 20 [deg] and the policy parameters to be optimized were the three-dimensional control gain vector and CPG parameters $\;\theta ={\left[k_{\varphi },k_{\dot{\varphi }},k_{\dot{\vartheta }},\omega ,\beta \right]}^{T}.$ Note that since ${\dot{\vartheta }}_{L},{\dot{\vartheta }}_{R}$ share the same control gain as $k_{\dot{\vartheta }}$ the feedback gain is given by $\text{K}={\left[k_{\varphi },k_{\dot{\varphi }},k_{\dot{\vartheta }L},k_{\dot{\vartheta }R}\right]}^{T}.$

The sampling rate was 0.005 [s] for each step, and one episode contained 12,000 steps (=60 [s]). The robot rested for 4 [s] at the end of each episode to recover its resting angle. We updated the hyperparameters every 10 episodes for 10 iterations, meaning 100 episodes for an entire run. The initializations of the hyperparameters were $\eta_{\text{0}}={\left[0,0,0,0,0\right]}^{T},$ and $\sigma_{\text{0}}={\left[100,200,20,20,20\right]}^{T}.$ The best PGPE learning rates were selected, respectively, from sets {10^−6^, 5 ⋅ 10^−6^, 10^−5^} and {10^−5^, 3 ⋅ 10^−5^, 5⋅10^−5^, 10^−4^}. Since EPHE-AB-mean was better than or equal to the other methods in all the three simulation results, we focused on a comparison between EPHE-AB-mean, (EPHE-AB in short in the following content) and PGPE with the best learning rate to investigate the different updating approaches of gradient estimating and reward weighting.

#### Results

We ran five independent runs and measured the average and the SE of the cumulative returns against the iterations of hyper parameter updating. An episode was regarded as successful if the agent bounced to the upright position and maintained its balancing until the episode’s end. Failures included when the agent could not stand up at all, swinging forward and backward at a relatively high speed, etc. We defined a successful learning as when the agent found the hyperparameters that achieved successful episodes within five iterations. The successful learnings of EPHE-AB and PGPE were 5/5 and 3/5, respectively.

**Figure 10:**
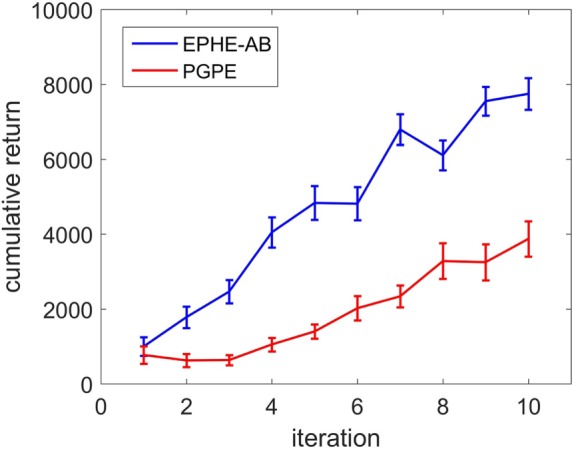
**Learning curves of EM-based Policy Hyperparameter Exploration-AB and PGPE in hardware experiments of standing up and balancing task**.

**Figure 11:**
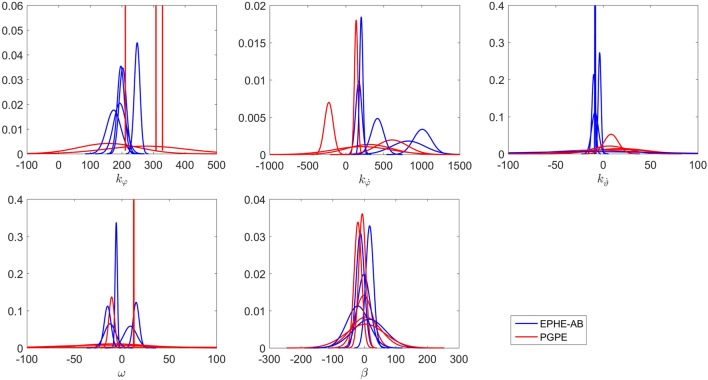
**Distributions of final parameters found by five runs of EM-based Policy Hyperparameter Exploration-AB and PGPE with best learning rate in hardware experiments of standing up and balancing task**.

**Figure 12:**
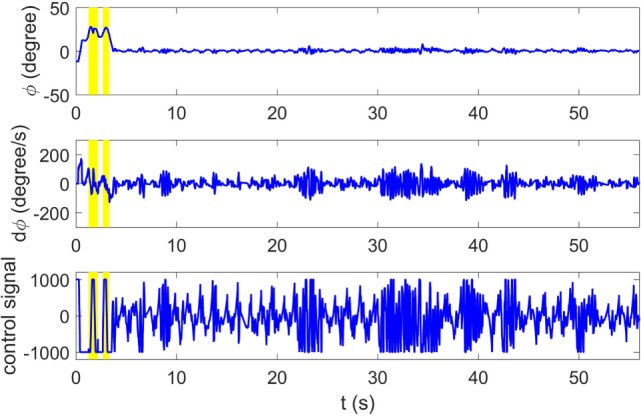
**Typical trajectory of states and control signal with switching controller optimized by EM-based Policy Hyperparameter Exploration-AB**.

Figure 10 compares the learning curves of EPHE-AB and PGPE with the best learning rate (α_η_ = 5 ⋅ 10^−6^, α_σ_ = 3 ⋅ 10^−5^). The error bars show 1 SD over five independent runs. Although PGPE learned successful behaviors, the successful rate was low. EPHE-AB outperformed PGPE during the whole learning process. Figure 11 shows the distributions of the final parameters found by five runs of EPHE-AB and PGPE with the best learning rate. With EPHE-AB, the parameters converged to similar distributions, while with PGPE, they often stayed flat or degenerated. For example, for key parameter *k*_φ_, EPHE-AB and PGPE found similar parameters ranging from 150 to 350, but only three of PGPE’s final distributions converged. For the second key parameter $k_{\dot{\varphi }},$ three of the PGPE distributions were near those successfully found by EPHE-AB but with larger variance, and two converged to the values away from the parameters found by EPHE-AB. Figure 12 shows the trajectory of a successful standing up and balancing task with one of the final hyper parameters: θ = [248.66, 1,007.23, −8.51, −5.85, 17.15] learned by EPHE-AB. The yellow area shows when the bumper was activated. The agent bounced three times, reached, and stayed in the target position after 4 s.

### Vision-Based Approaching Task

#### Experimental Setting

This task requires the smartphone balancer to learn to stand up and approach a visual target while balancing. The basic hardware setting is the same as in Section “Standing Up and Balancing Task.” We used an Android phone’s camera and detected a blob of a specified color and computed its center position and area using the OpenCV library. The state variables are $\text{x}={\left[\varphi ,\dot{\varphi },\;{\dot{\vartheta }}_{L},{\dot{\vartheta }}_{R},c_{x},D\right]}^{T},$ where *c_x_* ∈[−0.3, 0.3] is the blob center on the *X* axis, and *D* ∈[0, 10] is the distance to the target calculated by the inverse of the square root of the blob area $D=\frac{a}{\sqrt{S_{\text{blob}}}},$ where parameter *a* is set to scale *D* = 1–10 dm. When the target is invisible, we set *c_x_* = ±0.4 based on the previous sign of *c_x_*, and *D* = 15. When the target is visible, the immediate reward at time *t* is given by

$$r_{t}=\text{exp}\;\left(-w_{1}{\left(\frac{\varphi_{t}}{Z_{\varphi }}\right)}^{2}-w_{2}{\left(\frac{c_{x_{t}}}{Z_{c_{x}}}\right)}^{2}-w_{3}{\left(\frac{D_{t}-5}{Z_{D}}\right)}^{2}\right),$$

where $Z_{\varphi },\;Z_{c_{x}}$ and *Z_D_* are, respectively, the constants to normalize φ*_t_, c_xt_* (*D_t_* − 5) to [−1, 1]. We assigned *w*_1_ = *w*_2_ = 1.2, and *w*_3_ = 0.6. When the target was not visible, the reward was *r_t_* = 0. The return was computed by

$$R\left(h\right)=\sum_{t=1}^{T}r_{t},\;T=12,000.$$

**Figure 13:**
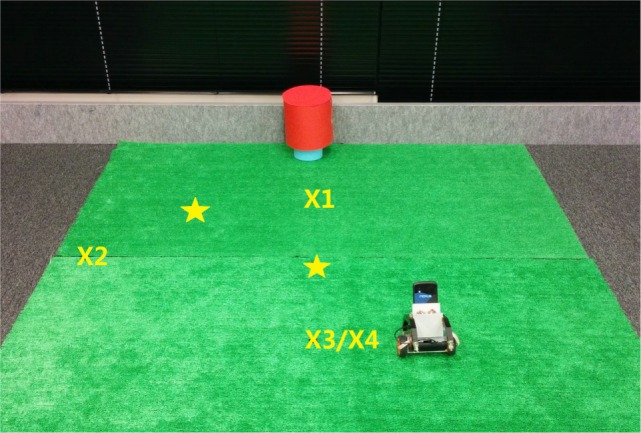
**Landscape and initial position of robot in approaching task**.

We set up four initial positions of the robot to start (Figure 13). The robot’s desired behavior is to start running from *D* = 10 (*X*_2_, *X*_3_, *X*_4_) or *D* = 2 (*X*_1_); it stops at *D* = 5 (star marks) while keeping the target in the middle and balancing. The robot lies down and faces straight ahead with initial state *X*_1_, *X*_2_, *X*_3_ and faces back to the target with initial state *X*_4_.

The following is the control structure:

When φ is inside the threshold φ_s_ = [−20°, 20°], and the linear feedback stabilizer is activated:

$$\begin{array}{llll} u_{L} & =-\left(k_{\varphi L}\varphi +k_{\dot{\varphi }L}\dot{\varphi }+k_{\dot{\vartheta }L}{\dot{\vartheta }}_{L}+\text{re}{\text{f}}_{L}+k_{\mathrm{cL}}c_{x}+k_{\mathrm{dL}}\left(D-5\right)\right)\; & & \;\\ u_{R} & =-\left(k_{\varphi R}\varphi +k_{\dot{\varphi }R}\dot{\varphi }+k_{\dot{\vartheta }R}{\dot{\vartheta }}_{R}+\text{re}{\text{f}}_{R}+k_{\mathrm{cR}}c_{x}+k_{\mathrm{dR}}\left(D-5\right)\right)\; & & \; \end{array}$$

Otherwise, the CPG-based destabilizer is activated as subsuper1.

The policy parameters are 14-dimensional

$$\theta \;=\;\left[k_{\varphi L},k_{\dot{\varphi }L},k_{\dot{\vartheta }L},k_{\varphi R},k_{\dot{\varphi }R},k_{\dot{\vartheta }R},\text{re}{\text{f}}_{L},\text{re}{\text{f}}_{R},k_{\mathrm{cL}},k_{\mathrm{dL}},k_{\mathrm{cR}},k_{\mathrm{dR}},\omega ,\beta \right]$$

$k_{\varphi L},k_{\dot{\varphi }L},k_{\dot{\vartheta }L},k_{\varphi R},k_{\dot{\varphi }R},k_{\dot{\vartheta }R}$ are the linear feedback control gains for stabilization, ref*_L_* and ref*_R_* are the reference constants for the wheel to move forward and backward, *k_cL_, k_dL_, k_cR_, k_dR_* are the control gain for the steering, and ω, β are the CPG parameters. The difference of *k_cL_* and *k_cR_* can cause rotation toward the target, but we set up all the parameters differently for the left and right wheels to adapt to any asymmetry in the hardware and the electronics. The sampling rate was 0.005 [s] for each step, and one episode was 12,000 steps (=60 [s]). We changed the initial position in every five episodes. The hyperparameters are updated every 20 episodes for 10 iterations, resulting in 200 episodes for 1 entire run.

The robot was forced to stop when *D* ≤ 1 for safety concerns. The initial hyper parameters were setup based on the optimal parameters we obtained from Section “Standing Up and Balancing Task”:

$$\begin{array}{llll} \eta_{\text{0}} & ={\left[250,1000,-9,250,1000,-9,200,200,0,0,0,0,-6,17\right]}^{T}\\ \sigma_{\text{0}} & ={[50,50,10,50,50,10,500,500,2,000,100,2,000,100,10,10]}^{T}.\; & & \; \end{array}$$

The best learning rates for PGPE were selected from sets {10^−6^, 10^−5^, 10^−4^} and {10^−6^, 10^−5^, 10^−4^}.

#### Results

We ran five independent runs and measured the average and the SE of the cumulative returns against the iterations of hyperparameter updating. An episode was regarded as successful if the robot could stand up regardless of its initial position and facing direction, move toward the target position, and keep balancing until the end of the episode. Failures included when the agent kept moving around and was unable to stand up when the target was spotted, or when it took too long to learn balancing instead of moving forward, etc. A successful learning was defined as when the agent identified the hyperparameters that achieved the successful episodes within five iterations. The successful learning of EPHE-AB and PGPE was 2/5 and 0/5, respectively.

**Figure 14:**
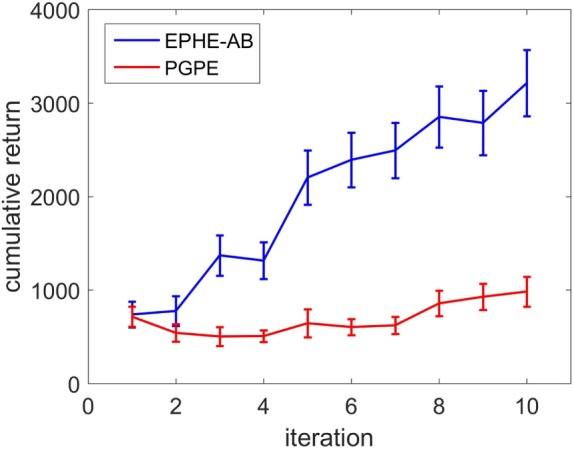
**Learning curve of EM-based Policy Hyperparameter Exploration-AB and PGPE in hardware experiments of vision based approaching task**.

Figure 14 shows the learning curves of EPHE-AB and PGPE with the best learning rate (α_η_ = 10^−5^, α_σ_ = 10^−5^). The error bars show 1 SD over five independent runs. PGPE failed to learn successful behaviors while EPHE-AB shows a clear learning curve.

**Figure 15:**
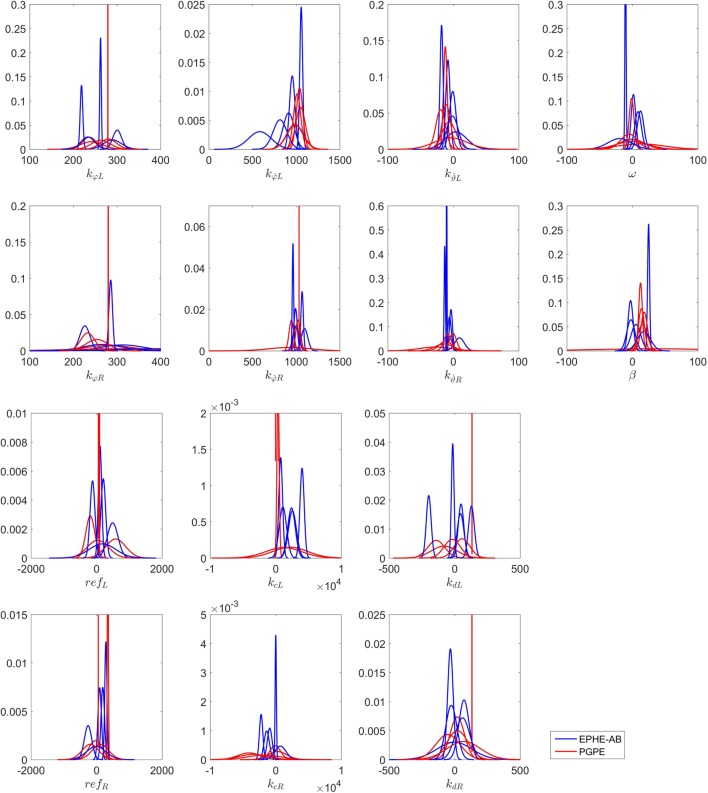
**Distributions of final parameters found by five runs of EM-based Policy Hyperparameter Exploration-AB and PGPE with best learning rate in hardware experiments of vision-based approaching task**.

Figure 15 shows the distributions of the final parameters found by EPHE-AB and PGPE with the best learning rate. Most of the parameters found by EPHE-AB converged, while with PGPE, even though each parameter has one or two convergent cases, the directions of the convergences were scattered. The key steering parameters in this task were *k_cL_* and *k_cR_*. The successful pairs found by EPHE-AB were {*k_cL_, k_cR_*} = {2,548, −1,377} and {1,092, −2,230}, while PGPE failed to discover similar pair values. Hence, it was difficult for the agent to spot the target, not to mention standing and approaching.

The figures in the Supplementary Material show the trajectories and video snapshots of the successful episodes with the parameters [290, 955, 4, 226, 1,096, 9, 204, 189, 1,092, 48, −2,230, −33, −11, 25] found by EPHE-AB from starting positions *X*_2_, *X*_4_, *X*_1_, and *X*_3_.

## Discussion

In Section “Method,” we compared EPHE-AB with the baseline of mean, EPHE-CW, EPHE-RW, EPHE-K, PGPE, NES, and FEM in three simulation tasks. EPHE-AB significantly outperformed the others in the cart-pole balancing and smartphone standing up tasks in the beginning of learning. In smartphone standing up task, EPHE with REPS Weighting achieved higher returns in the end of learning, this is due to the convergence of η to small value when most of the returns are close to 1,000. When η is small, it gives heavy weights to very small number of the highest returns.

We examined the learning behaviors of EPHE-ABs with different baselines, including the fixed baseline with half of the sample size, mean, and 1 and 2 SDs from the mean. We found no significant performance differences among all of the baselines in the swing up and cart-pole balancing tasks; however, the baseline with the mean outperformed the others in the smartphone standing up tasks. This result suggests that the baseline with the mean discarded most of the samples in the early stage of learning and led to a steady decrease of the number of discarded samples during learning. By examining the return distribution of different tasks, we found that the adaptive baseline plays an important role in the beginning of learning, when a relatively small number of episodes result in large returns. While in the end of learning, the mean baseline is lower than the fixed baseline when the returns contain several values around 0. This also explains the better performance of EPHE with REPS Weighting in the smartphone standing up task (see Figure S1 in Supplementary Material).

In the hardware experiments in Section “Hardware Experiments,” we compared EPHE-AB with the baseline of mean with PGPE with the best learning rate and showed that EPHE-AB learned the tasks more reliably and efficiently than PGPE.

In the Android-bot simulation task (see Standing Up and Balancing of Two-Wheeled Smartphone Robot), PGPE with the best learning rate performed worse than EPHEs. A possible reason is that, we used the same learning rate for updating all of the hyperparameters, even though the gradients for the mean and the variance of different parameters can be largely different. This reason might also have caused PGPE to be trapped in the local optima. In the hardware experiments, the results in Figure 10 showed that PGPE learned appropriate parameters with slow climbing. However, in a more complicated task, the results of Figure 14 showed that even with the best learning rate PGPE performed much worse than EPHE-AB. Figures 11 and 15 showed different situations of the convergence of EPHE-AB and PGPE based on the learned hyperparameters and provided a hint of the failures of PGPE resembled the smartphone simulation task. PGPE might work better if we introduced different learning rates for different hyperparameters, but that would necessitate more samples for reliable estimates of the optimal learning rates. On the other hand, EPHE-AB realized adaptive step size based on simple reward-weighted mean and variances of the sampled parameters. It could almost reach the optimum in the hyper parameter space after only one or two iterations while PGPE might move slowly in the wrong direction.

EM-based Policy Hyperparameter Exploration-AB achieved better performances in the hardware experiments. In the standing up and balancing tasks, it achieved a 100% successful rate. The vision-based approaching task was much more challenging not only because of its additional task requirement but also due to the various initial positions, which increased the variance in the performance evaluation. Nevertheless, EPHE-AB achieved a 40% success rate. By analyzing the learning history, we found that many of the episodes starting from the most difficult position *X*_4_ were discarded because of relatively low returns. A possible solution is to evaluate each sample of the parameters over multiple starting conditions.

PGPE worked much worse in the hardware experiments than in the simulation tasks. For the standing up and balancing task in the hardware, the wheel angle was not usable and we used a simple binary reward function might be the reason. In the approaching task, the increased number of policy parameters indicates more difficulties to PGPE. As illustrated in Figure 15, such hyperparameters for steering the robot as *k_cL_, k_dL_, k_cR_, k_dR_* in PGPE usually failed. Hence, the robot never fairly spotted the target, so that it obtained low rewards.

## Conclusion and Future Work

In this paper, we improved the updating mechanism of the EPHE method by computing an adaptive baseline (EPHE-AB) to discard inferior samples. Note that the adaptive baseline can be applied to the methods under the EM-based policy search framework. We verified the improvements in three simulation tasks, a pendulum swing up with limited torque, cart-pole balancing, and a simulator of our smartphone balancer that compared to EPHE-CW, EPHE-RW, EPHE-K, PGPE, and NES with the best learning rate and FEM. Our results showed that EPHE-AB achieved the best performance among the other methods in the beginning of learning when a fixed baseline is inadequate to preserve informative samples. The choice of the baseline of mean is more effective in focusing on positive outliers than others in the early stage of learning. This is important for learning from primitive initial parameters and random initial states. We also implemented EPHE-AB with the baseline of mean in our real smartphone robot system to achieve two tasks, standing up and balancing, and approaching a visual target while balancing. We compared EPHE-AB-mean with PGPE in hardware experiments. EPHE-AB-mean achieved nearly optimal behaviors in as few as five iterations in both tasks, demonstrating its efficient and tuning-free learning.

Note that in the episodic manner, many policy search methods derived from different principles are equivalent or similar, like EPHE-K, PoWER, and PI^2^, and EPHE-RW and REPS. In the future, we investigate the step-based EPHE for optimizing stochastic policies to reveal the difference between EPHE and the other algorithms. Since EPHE can use different probability distribution for policy parameters and hyperparameters, the different update rules can be derived in this case.

The EPHE methods with adaptive baseline were shown to be a promising approach in actual robotic tasks, but they discarded a part of the training samples based on the return values. To overcome this data inefficiency, a promising direction is the importance sampling technique that reuses samples over multiple iterations. Previous studies show that using an importance sampling technique achieves significant performance improvement in PGPE (Zhao et al., 2013) and RWR (Hachiya et al., 2011). EPHE-AB can be straightforwardly integrated with importance sampling by considering the ratio between parameter distributions in different iterations. This extension will be left for future study.

The smartphone robot project is developing a low-cost and high-performance robotic platform to form a sustainable robot colony, where individuals can achieve self-preservation and self-reproduction behaviors (Doya and Uchibe, 2005). With a two-wheeled balancer, we utilized a smartphone’s multiple sensors and achieved such basic behaviors as standing up, balancing, and approaching. For the next stage, we will continue developing reinforcement learning algorithms to achieve foraging, homing behaviors of single agents, and communication among multiple agents to form an adaptive, autonomous robotic colony.

## Author Contributions

JW, EU, and KD conceived the method. JW performed experiments and analyzed data. JW, EU, and KD wrote the paper.

## Conflict of Interest Statement

The authors declare that the research was conducted in the absence of any commercial or financial relationships that could be construed as a potential conflict of interest.

## Supplementary Material

Video S1 **Learning behaviors of smartphone balancer**.

## Appendix

### A.1 Robot Construction

#### Hardware Components and Connection

As the balancer’s brain, we used Nexus 4, which was co-developed by Google and LG in November 2012. It has a 1.512-GHz quad-core Krait CPU, 2 GB RAM, and is operated on an Android Jelly Bean 4.2 system, which is compatible with IOIO OTG USB in the debugging mode. IOIO OTG is a board that provides a host machine that can interface with external hardware over various commonly used protocols. Since it was specifically designed to work with Android devices, programing can only be done on the Android side with IOIO libraries. We used the HUB-EE wheel as a robot servo. It integrates a DC motor, a Toshiba TB6593FNG motor driver, and a quadrature encoder, for real-time data recovery. AutoCAD was used to design the chassis, and Ultimaker 2 was used to print out each bit of the chassis.

Figure S6 in Supplementary Material shows the hardware’s connection. Nexus 4 is connected to IOIO through a USB cable. The battery outputs 5 V into the IOIO board to supply power to both the IOIO board and the smartphone. The IOIO board also provides 5 V to trigger the HUB-EE wheel. The connect break-out board clarifies communication channel and provides a neat socket connection between the HUB-EE wheel and the IOIO board.

#### Software Setup

The program was written in Java by developing an Android app under Android Studio. The IOIO developer provides high level Java APIs including IOIOLib Core, and IOIOLib Application to read in and out the digital input/output and the PWM input/output to transfer the sensory information and control signals. We also used the Open CV library to capture the target’s visual information. In the standing up and balancing task, three threads work simultaneously: the IOIO thread that sends control signals and retrieves encoder data every 1 ms (control cycle), a sensor thread that retrieves a fused gyro and an accelerometer every 6 ms, and a UI thread that visualize the robot’s state and its learning process. In the approaching task, the camera sensor is activated and updated every 30 ms.

### A.2 Simulation Setup

The equation of motion of the inverted pendulum task in Section “Pendulum Swing Up with Limited Torque” was given by

$$ml^{2}\ddot{\varphi }=-\mu \ddot{\varphi }+\mathrm{mgl}\sin\;\varphi +u$$

where *m* = 1 [kg] is the pendulum mass, *l* = 1 [m] is the pendulum length, μ = 0.01 [kg m^2^/s] is the coefficient of friction, and *g* = 9.81 [m/s^2^] is the gravitational constant.

The equation of motion of the cart-pole balancing task in Section “Cart-Pole Balancing” was given by

$$\begin{array}{llll} ẍ & =\frac{F-m_{p}l\left(\ddot{\varphi }\;\cos\;\varphi -{\ddot{\text{φ}}}^{2}\sin\;\varphi \right)}{m_{c}+m_{p}}\; & & \;\\ \ddot{\varphi } & =\frac{g\sin\;\varphi \left(m_{c}+m_{p}\right)-\left(F+m_{s}l{\ddot{\text{φ}}}^{2}\sin\;\varphi \right)\cos\;\varphi }{\frac{4}{3}l\left(m_{c}+m_{p}\right)-m_{p}l\cos^{2}\varphi }\; & & \; \end{array}$$

where *m_c_* = 1 [kg] is the cart mass, *m_p_* = 0.1 [kg] is the pole mass, *l* = 0.5 [m] is the half length of the pole, *g* = 9.81 [m/s^s^] is the gravitational constant and −10*N* ≤ *F* ≤ + 10*N* is the force applied to the cart.

The equation of motion of the two-wheeled smartphone robot in Section “Standing Up and Balancing of Two-Wheeled Smartphone Robot” was given by

$$\begin{array}{llll} \dot{\vartheta } & =\left[\left(\alpha +m_{p}\mathrm{lr}\;\cos\;\varphi \right)\left(T_{R}+T_{L}\right)+m_{p}^{2}l^{2}\mathrm{rg}\;\sin\;\varphi \;\cos\;\varphi \right.\; & & \;\\ & \;-{\text{m}}_{\text{p}}\mathrm{lr}\alpha {\ddot{\varphi }}^{2}\sin\;\varphi -m_{p}\mathrm{lr}\;\sin\;\varphi \;\cos\;\varphi F_{s}h\; & & \;\\ & \;\left.-2b\dot{\vartheta }\left(m_{p}\mathrm{lr}\;\cos\;\varphi +\alpha \right)\right]∕\left[\alpha \beta +m_{p}^{2}l^{2}r^{2}\cos^{2}\varphi \right]\; & & \;\\ \ddot{\varphi } & =\left[\left(\beta -m_{p}\mathrm{lr}\;\cos\;\varphi \right)\left(T_{R}+T_{L}\right)+m_{p}^{2}l^{2}r^{2}{\ddot{\varphi }}^{2}\sin\;\varphi \;\cos\;\varphi \right.\; & & \;\\ & \;+m_{p}l\beta g\;\sin\;\varphi -\beta \;\sin\;\varphi F_{s}h+2b\overset{¨}{\vartheta }\; & & \;\\ & \;\;\;\;\;\;\left.\times \left(m_{p}\mathrm{lr}\;\cos\;\varphi -\beta \right)\right]∕\left[\alpha \beta +m_{p}^{2}l^{2}r^{2}\cos^{2}\varphi \right]\; & & \; \end{array}$$

where

$$\begin{array}{llll} \alpha & =I_{p}+m_{p}l^{2}\; & & \;\\ \beta & =2I_{w}-m_{p}r^{2}-2m_{c}r^{2}\; & & \; \end{array}$$

Specifically, the spring force is derived by Hooke’s law:

$$F_{s}=k\Delta L+c\ddot{\varphi }=\mathrm{kh}\left(\cos\;\varphi_{0}-\cos\;\varphi \right)+c\ddot{\text{φ}}$$

where *m_p_* = 0.354 [kg] is the robot body mass, *m_w_* = 0.024 [kg] is the single wheel mass, *l* = 0.06 [m] is the length from the center of the robot body, *r* = 0.03 [m] is the wheel’s radius, $I_{w}=\frac{1}{2}m_{w}r^{2}\;\left[\text{kg}{\text{m}}^{2}\right]$ is the moment of inertia of the wheel, $I_{p}=\frac{4}{3}m_{p}l^{2}\;\left[\text{kg}{\text{m}}^{2}\right]$ is the moment of inertia of the robot body, *g* = 9.81 [m/s^2^] is the gravity acceleration, *c* = 0.05 [N^2^/s] is the damper coefficient of the spring, *k* = 0.8869 [N/mm] is the spring coefficient, *h* = 0.096 [m] is the spring location along the robot body, φ_0_ is the threshold angle for the activating spring, *b* = 0.0001 [kg m^2^/s] is the friction coefficient of the wheel axle, and *T_R_* and *T_L_* are the applied torque to the right and left wheels, respectively.

## References

[B1] Abdolmaleki A. Lau N. Reis L. P. Neumann G. (2015). “Regularized covariance estimation for weighted maximum likelihood policy search methods,” in 2015 IEEE-RAS 15th International Conference on Humanoid Robots (Humanoids) (Seoul: IEEE), 154–159.

[B2] Deisenroth M. P. Neumann G. Peters J. (2013). A survey on policy search for robotics. Found. Trends Rob. 2, 1–142. 10.1561/2300000021

[B3] Doya K. (2000). Reinforcement learning in continuous time and space. Neural Comput. 12, 219–245. 10.1162/089976600300015961 10636940

[B4] Doya K. Uchibe E. (2005). The Cyber rodent project: exploration of adaptive mechanisms for self-preservation and self-reproduction. Adapt. Behav. 13, 149–160. 10.1177/105971230501300206

[B5] Hachiya H. Peters J. Sugiyama M. (2011). Reward-weighted regression with sample reuse for direct policy search in reinforcement learning. Neural Comput. 23, 2798–2832. 10.1162/NECO_a_00199 21851281

[B6] Hansen N. Ostermeier A. (2001). Completely derandomized self- adaptation in evolution strategies. Evol. Comput. 9, 159–195. 10.1162/106365601750190398 11382355

[B7] Kober J. Peters J. (2011). Policy search for motor primitives in robotics. Mach. Learn. 84, 171–203. 10.1007/s10994-010-5223-6

[B8] Kupcsik A. G. Deisenroth M. P. Peters J. Neumann G. (2013). “Data-efficient generalization of robot skills with contextual policy search,” in Proceedings of the AAAI Conference on Artificial Intelligence, Bellevue.

[B9] Mannor S. Rubinstein R. Y. Gat Y. (2003). “The cross-entropy method for fast policy search,” in Proc. of the 20th International Conference on Machine Learning, Washington, DC.

[B10] Peters J. Katharina M. Yasemin A. (2010). “Relative entropy policy search,” in Proceedings of the AAAI Conference on Artificial Intelligence, Atlanta.

[B11] Peters J. Schaal S. (2007). “Reinforcement learning by reward-weighted regression for operational space control,” in International Conference on Machine Learning, Corvallis.

[B12] Riedmiller M. Peters J. Schaal S. (2007). “Evaluation of policy gradient methods and variants on the cart-pole benchmark,” in IEEE Symposium on Approximate Dynamic Programming and Reinforcement Learning, Honolulu.

[B13] Sehnke F. Osendorfer C. Rückstieß T. Graves A. Peters J. Schmidhuber J. (2010). Parameter-exploring policy gradients. Neural Networks 21, 551–559. 10.1016/j.neunet.2009.12.004 20061118

[B14] Silver D. Lever G. Heess N. Degris T. Wierstra D. Riedmiller M. (2014). “Deterministic policy gradient algorithms,” in Proc. of 31st International Conference on Machine Learning, Beijing.

[B15] Stulp F. Sigaud O. (2012). “Path integral policy improvement with covariance matrix adaptation,” in Proc. of the 29th International Conference on Machine Learning, (Edinburgh), 281–288.

[B16] Theodorou E. Buchli J. Schaal S. (2010). A generalized path integral control approach to reinforcement learning. J. Mach. Learn. Res. 11, 3137–3181.

[B17] Wang J. Uchibe E. Doya K. (2013). Standing-Up and Balancing Behaviors of Android Phone Robot. Hongkong: Technical Committee on Nonlinear Problems, IEICE.

[B18] Wang J. Uchibe E. Doya K. (2014). “Control of two-wheel balancing and standing-up behaviors by an android phone robot,” in Annual Conference on Robotics Society of Japan – RSJ, Fukuoka.

[B19] Wang J. Uchibe E. Doya K. (2016). EM-based policy hyper parameter exploration: application to standing and balancing of a two-wheeled smartphone robot. Artif. Life Rob. 21, 125–131. 10.1007/s10015-015-0260-7

[B20] Wierstra D. Schaul T. Glasmachers T. Sun Y. Peters J. Schmidhuber J. (2014). Natural evolution strategies. J. Mach. Learn. Res. 15, 949–980.

[B21] Wierstra D. Schaul T. Peters J. Schmidhuber J. (2008). “Fitness expectation maximization,” in Proceedings of Parallel Problem Solving from Nature (PPSN) (Dortmund).

[B22] Williams R. J. (1992). Simple statistical gradient-following algorithms for connectionist reinforcement learning. Mach. Learn. 8, 229–256. 10.1023/A:1022672621406

[B23] Yoshida N. Yoshimoto J. Uchibe E. Doya K. (2012). “Development of robot platform with smart phone,” in The Annual Conference on Robotics Society of Japan – RSJ, Sapporo.

[B24] Zhao T. Hachiya H. Niu G. Sugiyama M. (2012). Analysis and improvement of policy gradient estimation. Neural Networks 26, 118–129. 10.1016/j.neunet.2011.09.005 22019189

[B25] Zhao T. Hachiya H. Tangkaratt V. Morimoto J. Sugiyama M. (2013). Efficient sample reuse in policy gradients with parameter-based exploration. Neural Comput. 25, 1512–1547. 10.1162/NECO_a_00452 23517103

