# Supplementary material for: Adaptive Baseline Enhances EM-Based Policy Search: Validation in a View-Based Positioning Task of a Smartphone Balancer
Source: Front Neurorobot. 2017 Jan 23;11:1. doi: 10.3389/fnbot.2017.00001 (PMC5256123; doi:10.3389/fnbot.2017.00001)
Supplement: Supplementary file 1 [file Presentation_1.PDF]

Supplementary Materials: Adaptive Baseline Enhances EM-based Policy Search: Validation in a View-based Positioning Task of a Smartphone Balancer

Return distributions of one successful learning in a standing-up and balancing simulation of smartphone robot task

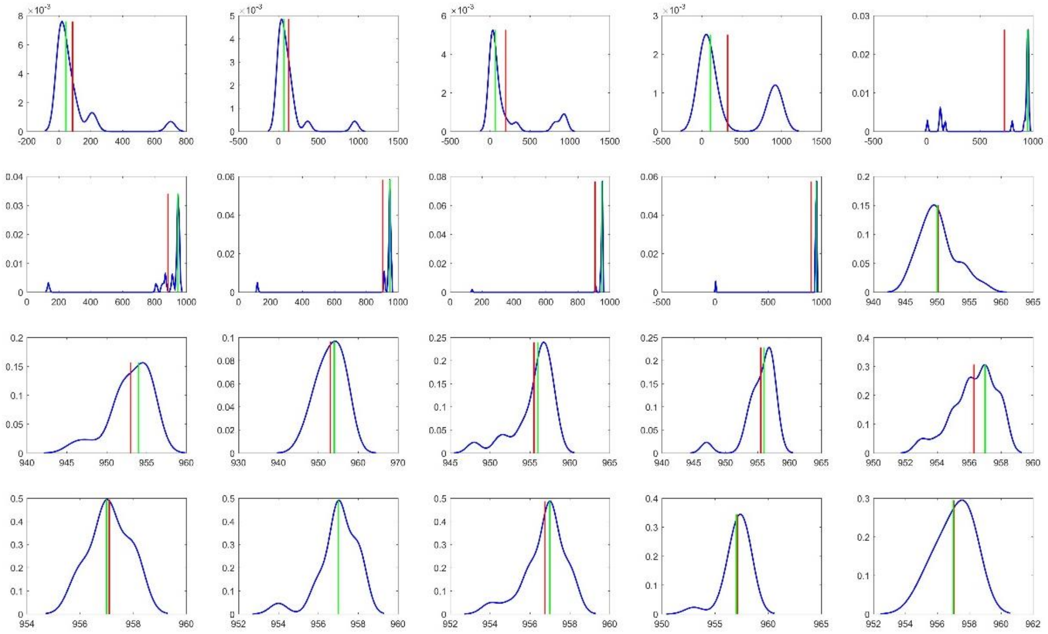

Figure S1 Return distributions of one successful learning in a standing-up and balancing simulation of smartphone robot task

Figure S1 shows the return distribution of one successful learning in smartphone balancer simulation task with 20 samples in each iteration from iteration 1 to iteration 20. The red line suggests the adaptive baseline and the green line suggests the fixed baseline of selected sample size equals 10. Note that we use ksdensity function in MATLAB to calculate the distribution.

Dynamics and Video Snapshot from X2 and X4 in Approaching Task

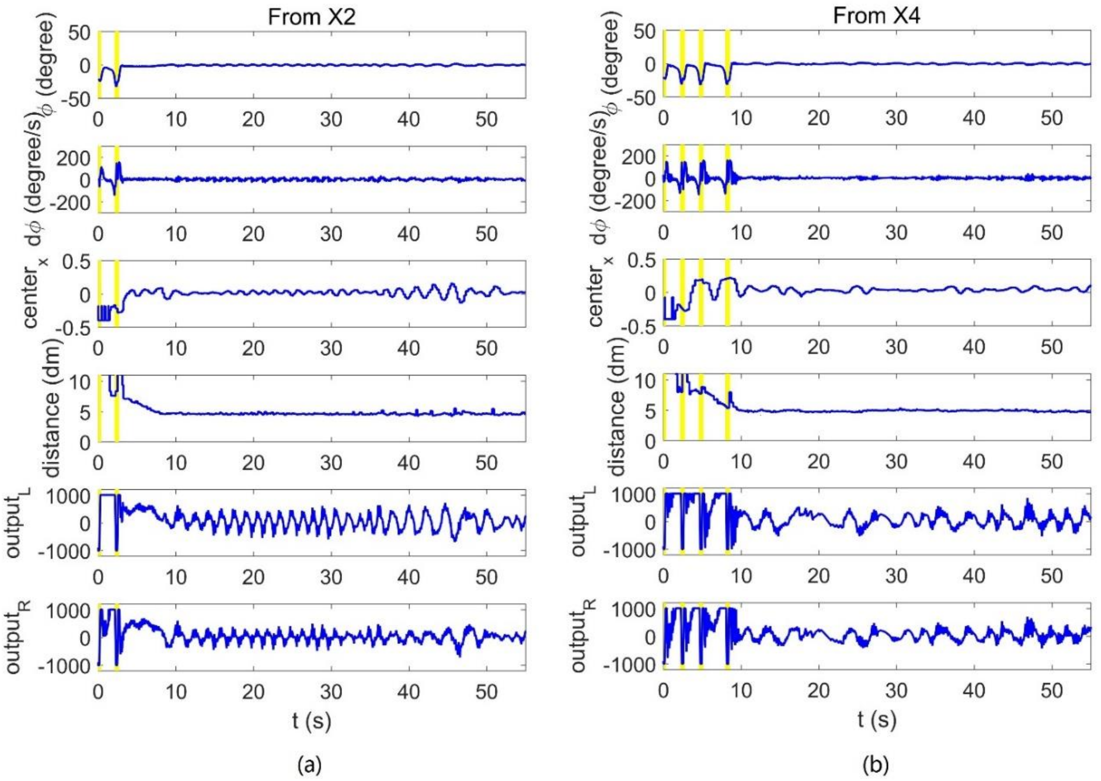

Figure S2 Trajectories of successful episodes from start positions X2 (a) and X4 (b)

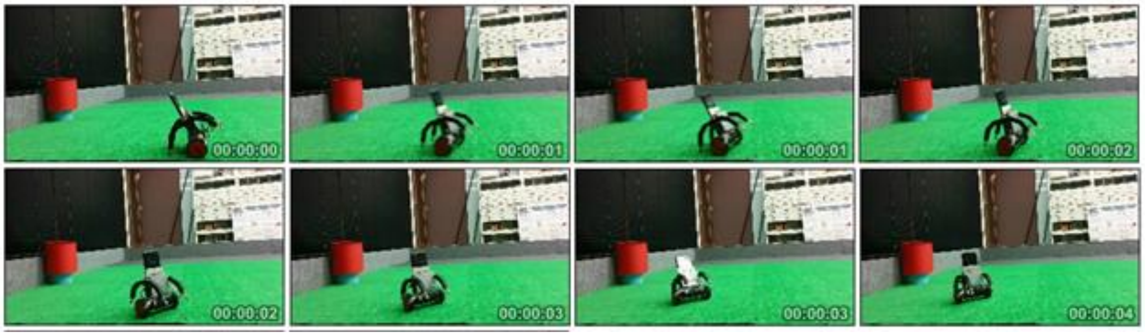

From X2 (a)

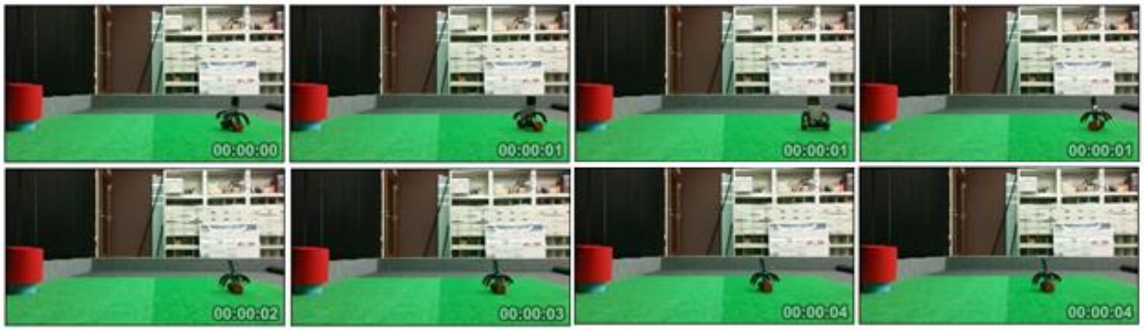

From X4 (b)

Figure S3 Video snapshot of successful behaviors from X2 (a) and X4 (b)

Yellow area shows when the bumper is activated. From X2 and X4, the robot initially did not face the target, so it circled until it found the target. From X2, it took 3 seconds to navigate and 5 seconds to approach while it bounced twice to stand up. From X4, the agent faced back to the target. It took around 4 seconds for navigation and around 6 seconds for approaching. It bounced four times to stand up. Note that there is time difference between the system and video record.

Dynamics and Video Snapshot from X1 and X3 in Approaching Task

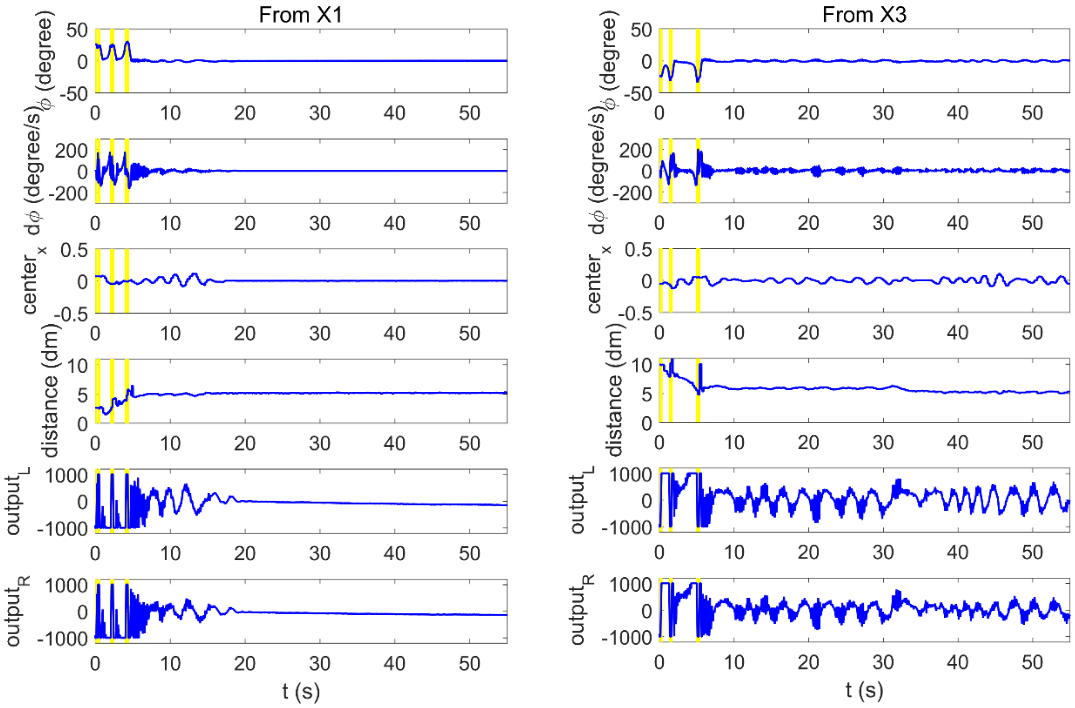

Figure S4 Trajectories of successful episodes from start positions X1 and X3

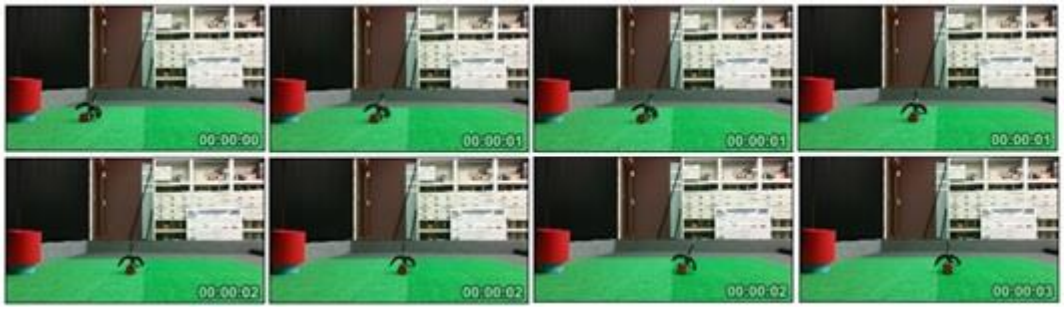

From X1

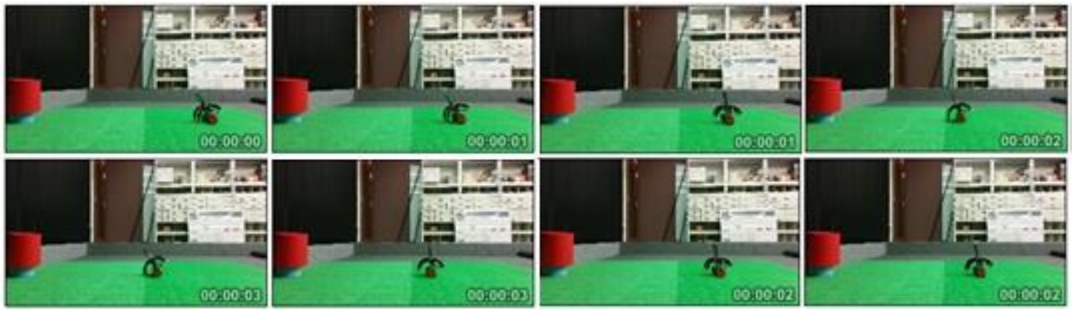

From X3

Figure S5 Video snapshot of successful behaviors from X1 and X3

Robot hardware connection

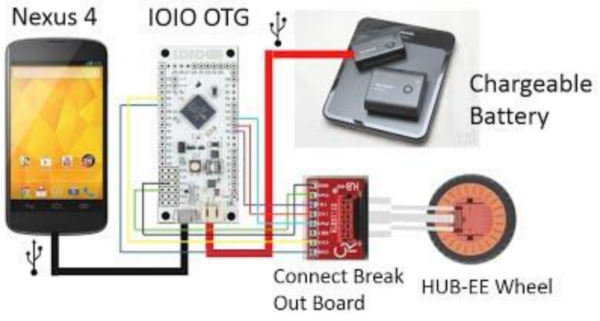

Figure S6 Hardware connection
